# Risk stratification of indeterminate thyroid nodules by novel multigene testing: a study of Asians with a high risk of malignancy

**DOI:** 10.1002/1878-0261.13205

**Published:** 2022-03-12

**Authors:** Chunfang Hu, Weiwei Jing, Qing Chang, Zhihui Zhang, Zhenrong Liu, Jian Cao, Linlin Zhao, Yue Sun, Cong Wang, Huan Zhao, Ting Xiao, Huiqin Guo

**Affiliations:** ^1^ Department of Pathology National Cancer Center/National Clinical Research Center for Cancer/Cancer Hospital Chinese Academy of Medical Sciences and Peking Union Medical College Beijing China; ^2^ Department of Ultrasound National Cancer Center/National Clinical Research Center for Cancer/Cancer Hospital Chinese Academy of Medical Sciences and Peking Union Medical College Beijing China; ^3^ State Key Laboratory of Molecular Oncology Department of Etiology and Carcinogenesis National Cancer Center/National Clinical Research Center for Cancer/Cancer Hospital Chinese Academy of Medical Sciences and Peking Union Medical College Beijing China

**Keywords:** DNA‐RNA test, indeterminate thyroid nodules, risk stratification, RNA test, rule‐in, rule‐out

## Abstract

Molecular testing of indeterminate thyroid nodules informs about the presence of point mutations, insertions/deletions, copy number variants, RNA fusions, transcript alterations and miRNA expression. American Thyroid Association (ATA) guidelines suggest molecular testing of indeterminate thyroid nodules may be considered to supplement risk of malignancy (ROM). Although these recommendations have been incorporated in clinical practices in the United States, molecular testing of indeterminate thyroid nodules is not common practice in Asia. Here, we performed molecular testing of 140 indeterminate nodules from Chinese patients using a novel molecular platform composed of RNA and DNA‐RNA classifiers, which is similar to Afirma GEC and ThyroSeq v3. Compared with reports from North America, the new RNA and DNA‐RNA classifiers had a higher positive predictive value (p1 = 0.000 and p2 = 0.020) but a lower negative predictive value (p1 = 0.004 and p2 = 0.098), with no significant differences in sensitivity (p1 = 0.625 and p2 = 0.179) or specificity (p1 = 0.391 and p2 = 0.264). Out of 58 resected nodules, 10 were borderline and 33 malignant, indicating a 74.1% ROM, which was higher than reports in North America (10–40% ROM). Our findings emphasize molecular testing with the newly reported RNA and DNA‐RNA classifiers can be used as a ‘rule‐in’ test when ROM is high.

## Graphical abstract

About 20% of thyroid cytology findings are indeterminate, and molecular testing can be used for risk stratification. Here, we developed a novel molecular platform and performed molecular testing of indeterminate thyroid nodules. Our findings emphasize that molecular testing with the newly reported RNA and DNA‐RNA classifiers has a high positive predictive value and can be used as a ‘rule‐in’ test in Asia.

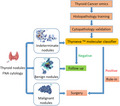

**Abbreviations**
ATA: American Thyroid Association
AUS/FLUS: atypia of undetermined significance/follicular lesion of undetermined significance
FN/SFN: follicular neoplasm/suspicious for a follicular neoplasm
FNA: fine‐needle aspiration
NIFTP: non‐invasive follicular thyroid neoplasm with papillary‐like nuclear features
NPV: negative predictive value
PPV: positive predictive value
ROM: risk of malignancy
TBSRTC: The Bethesda System for Reporting Thyroid Cytopathology
WT‐UMP: well‐differentiated tumour of uncertain malignancy

## 1 Introduction

In the last three decades, the number of cases of thyroid cancer has increased, becoming the most common type of cancer worldwide [1]. In the evaluation of preoperative thyroid nodules, traditional ultrasound‐guided fine‐needle aspiration (FNA) followed by cytological analysis is the most effective modality for distinguishing a malignant or benign nodule based on the Bethesda criteria [2, 3]. However, 20% of cases are indeterminate, including atypia of undetermined significance (AUS)/follicular lesion of undetermined significance (FLUS) (Bethesda category III) and follicular neoplasm (FN)/suspicious for a follicular neoplasm (SFN) (Bethesda category IV) [4]. Given the indeterminate diagnostic situation, the American Thyroid Association (ATA) suggests that molecular testing may be considered when assessing the malignancy risk of thyroid indeterminate nodules [5].

Molecular alterations in indeterminate thyroid nodules can be identified via various molecular detection platforms, including point mutations, insertions/deletions, copy number variants, RNA fusions, transcript alterations and miRNA expression. The commercial Afirma platform quantifies RNA expression alterations and applies an expression classifier; ThyroSeq v3 determines both DNA and RNA alterations. In addition, ThyraMIR uses a microRNA risk classifier [6, 7, 8, 9]. To date, Afirma and ThyroSeq are the most extensively used molecular platforms for molecular testing of thyroid nodules [10]. More importantly, from the original Afirma Gene Expression Classifier (GEC) to the current Afirma Gene Sequencing Classifier (GSC), the platform of Afirma is highlighted as a ‘rule‐out’ test for indeterminate thyroid nodules. A ‘rule‐out’ test implies that cytologically indeterminate nodules with negative Afirma results can be managed by clinical observation rather than diagnostic lobectomy [11, 12, 13]. In recent years, however, Afirma GECs have been replaced by a GSC approach in the United States and GSC is moving towards combined ‘rule‐in’ and ‘rule‐out’ uses. Regardless, ThyroSeq involves a seven‐gene panel test with high positive predictive value (PPV) (97%) and is regarded as a ‘rule‐in’ test [14], suggesting that cytologically indeterminate nodules with positive molecular results should be managed for surgery. Recently, the latest ThyroSeq v3 version of the 112‐gene panel has increased the negative predictive value (NPV) (90.9–97%) of the test, and combined ‘rule‐in’ and ‘rule‐out’ uses, in line with the Afirma GSC platform, are being used [14, 15, 16]. Of note, most studies of the two popular molecular platforms, Afirma and ThyroSeq v3, are based on populations from North America with a low risk of malignancy (ROM) of 10–40% in indeterminate categories [11, 12]. As such, the approach to molecular testing in North America may not be applicable in other regions of the world with different ethnicities and cultures.

There is increasing evidence concerning differences in the clinical practice of thyroid nodule management between Eastern and Western countries. Unlike Western countries, where diagnostic surgery is the long‐established standard of care for all FN/SFN nodules [17], strict triage for patients with indeterminate nodules for surgery is preferred in Asian countries. Indeed, a decision in favour of surgery would only be made when clinical/radiological examination revealed malignancy and the patient agreed to undergo surgery, ultimately resulting in a lower resection rate (RR) of indeterminate categories and increasing the ROM of resected indeterminate nodules in Asia [18, 19, 20]. Therefore, we sought to determine what role molecular testing will play in clinical practice in Asia.

However, neither of the two commercial molecular tests approved by the US Food and Drug Administration (FDA) is to date approved or available in China. To stratify indeterminate thyroid nodules, we designed a panel of multigene tests and successfully established a novel molecular platform based on data from a public database and 293 Chinese thyroid carcinoma samples. Our special panel involves a DNA‐ and RNA‐based next generation sequencing assay analysing 48 genes with a variety of genetic alterations, including point mutations, insertions/deletions and fusions, and expression profiles of 156 genes. Two classifiers were established based on the results of molecular testing: an RNA classifier similar to Afirma GEC and a DNA‐RNA classifier similar to ThyroSeq v3.

In the current study, we performed novel molecular testing of 140 indeterminate thyroid nodules from Chinese patients to enrich clinical practice data for molecular testing in regions outside North America.

## 2 Methods

### 2.1 Case selection

All patients who underwent thyroid FNA in the National Cancer Center between October 2018 and August 2019 were eligible for enrolment in this study. The study protocol was reviewed and approved by the Ethics Committee of the National Cancer Center/National Clinical Research Center for Cancer/Cancer Hospital (Reference Number: NCC2019C‐018), and written informed consent was obtained from each individual enrolled in this study. The study methodologies conformed to the standards set by the Declaration of Helsinki. Patients were selected on the basis of the following criteria: (a) cytological diagnosis of Bethesda III or Bethesda IV according to The Bethesda System for Reporting Thyroid Cytopathology (TBSRTC); and (b) at least 10 mL of PreservCyt^®^ (Hologic Inc., Marlborough, MA, USA) solution remaining after routine ThinPrep^®^ (Hologic Inc.) preparation. All patients signed informed consent before FNA.

Fine‐needle aspiration specimens were prepared as slides with ThinPrep^®^ 2000 (Hologic Inc.). Residual cells of cytologically indeterminate nodules (Bethesda category III or IV) were transferred to RNA Later within 48 h of ThinPrep^®^ preparation and sent for molecular testing, which was performed blindly without any patient information.

Cases of indeterminate cytology were managed based on ultrasound characteristics, clinical features and patient wishes, regardless of the results of molecular testing. The histopathology of surgical specimens was reviewed by pathologists who were blinded to the molecular testing results. All cases with a borderline diagnosis were re‐evaluated by a thyroid pathologist.

### 2.2 Development of the molecular testing platform

**Fig. 1:**
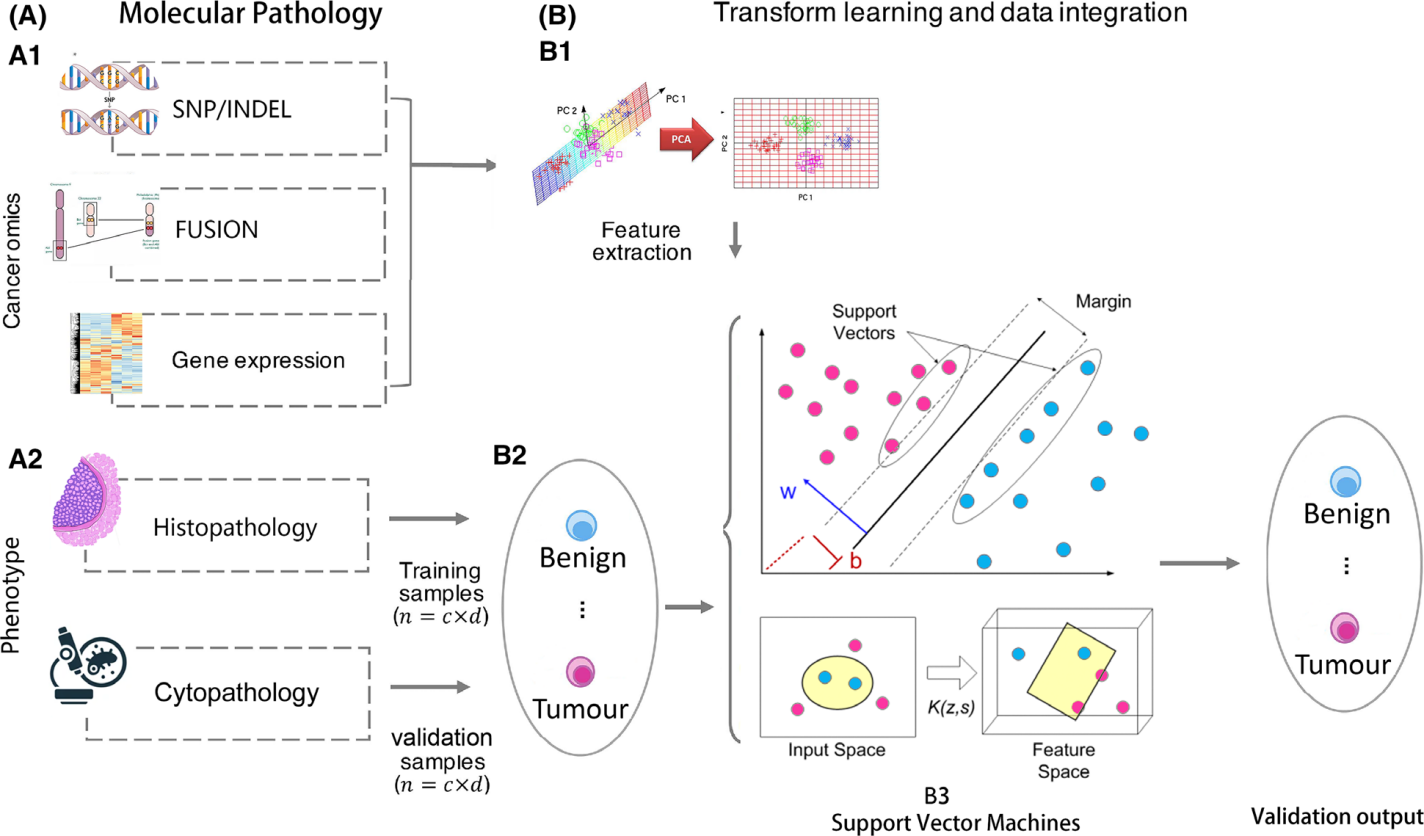
Development of the molecular platform. (A) The phenotype (including cytopathology and histopathology) and molecular results (including SNP/INDEL, fusion and expression) of test samples were integrated. (B) A kernel SVM model was proposed for machine learning of disease diagnosis based on integrated features. (B1) The process of transforming raw data into features that better represent the underlying problem to the predictive models resulted in improved model accuracy for unseen data. (B2) The histopathology of the samples was used as the SVM label; the cytopathology of the samples was used for validation. (B3) A Support vector machine classifier.

To distinguish malignant from benign thyroid nodules, we developed a machine learning model named Thyroeva™. As illustrated in Fig. 1, an SVM classifier model was constructed based on features of thyroid‐related gene mutation, fusion/deletion and expression, as derived from public data (TCGA/COSMIC/Arraymap/OncoKB) as well as 293 Chinese thyroid carcinoma tissues and corresponding adjacent normal tissues. The extracted features were next used for model training and optimization with 280 thyroid tissue samples. Finally, 64 thyroid nodules cytologically diagnosed as Bethesda category III/IV were used to verify the performance of the model, with histological diagnosis used as the gold standard. The sensitivity, specificity, accuracy and area under the ROC curve (AUC) of Thyroeva™ were 96% (95% CI: 90–99%), 93% (95% CI: 88–97%), 95% (95% CI: 90–100%) and 94% (95% CI: 89–99%) respectively.

### 2.3 DNA/RNA extraction

Genomic DNA was extracted using the Mag‐Bind Blood & Tissue DNA HDQ 96 kit (Omega Bioservices, Norcross, GA, USA). RNA was extracted using the Mag‐Bind Total RNA 96 kit (Omega Bioservices), and reverse transcription was performed with RevertAid First Strand cDNA Synthesis Kit (Thermo Fisher Scientific, Waltham, MA, USA). DNA/RNA purity was detected by a UV spectrophotometer (Nano Drop Technologies, Wilmington, DE, USA), and quantification was performed using qubit 3.0 (Fluorometer, Life Technologies, Carlsbad, California, USA).

### 2.4 Library preparation

A library was prepared using the Thyroeva™ (Shanghai Uthyroid Medical Technology Co., Ltd, Shanghai, China) panel, with three separate multiplex PCR amplification reactions: DNA (140 amplicons), RNA fusion (36 amplicons) and mRNA (169 amplicons) [21, 22]. DNA/ctDNA (20–200 ng) was added to each of the three reactions. After PCR and purification, the barcode and adaptors for NextSeq (Illumina, San Diego, CA, USA) were added, and the quality of the prepared libraries was assessed with LabChip GX Touch24 (PerkinElmer) [23]. Sequencing was carried out according to Illumina's protocols (Illumina) using a NextSeqCN500 (BerryGenomics, Beijing, China) with a 300‐cycle reagent cartridge corresponding to a 2 × 150 bp paired‐end configuration and with an average depth over 500×.

### 2.5 Sequencing data analysis

The DNA sequencing data were aligned to the human genome (hg19) with burrows–wheeler aligner 0.7.10 (Slashdot Media, San Diego, CA, USA). Local realignment and variant calling were performed with gatk v3.2‐2 (Broad Institute, Cambridge, MA, USA). To avoid false positive mutation calls arising due to DNA damage, calling thresholds of different mutations were applied to DNA samples of different quality. Variants with population frequencies > 0.1% in the ExAC, 1000 Genomes, dbSNP and ESP6500SI‐V2 databases were grouped as common SNPs and removed. Integrative Genomics Viewer (Broad Institute, Cambridge, MA, USA) was used to visualize variants aligned against the reference genome to confirm the accuracy of the variant calls by evaluating possible strand bias and sequencing errors. The RNA sequencing data were aligned to target amplicons with burrows–wheeler aligner 0.7.10 (Slashdot Media, San Diego, CA, USA), and in‐house‐developed software was used for subsequent data processing.

### 2.6 Interpretation of molecular testing results

The results of molecular testing were divided into three parts: DNA mutations, fusions and expression. A gene expression classifier was built based on RNA expression results and applied to assign a percentage value to each genetic alteration depending on the strength of the association with malignancy ≤ 10% (no association with cancer or low cancer probability) or > 10% (high cancer probability). In addition to the gene expression classifier, we trained a genomic classifier (GC) for all combined DNA and RNA alterations. The sum of the individual values for all detected alterations was calculated for the GC score, whereby ≤ 70% accepted was negative and > 70% was positive.

### 2.7 Statistics

Histopathology was taken as the gold standard for malignancy. Histopathological borderline lesions, including non‐invasive follicular thyroid neoplasms with papillary‐like nuclear features (NIFTPs) and well‐differentiated tumours of uncertain malignancy (WT‐UMP), were considered malignant because they represent a pre‐malignant entity that should be managed surgically. An analysis considering borderline lesions as benign is provided in Table S1.

Cytological and molecular testing results were compared with histopathological results, and the sensitivity, specificity, PPV and NPV of each test were calculated with 95% Wilson confidence intervals. Performance comparisons between the RNA classifier or DNA‐RNA classifier and their similar classifiers, namely, Afirma GEC and ThyroSeq v3, were carried out using the chi‐squared test. A chi‐squared test was also used to compare the diagnostic performance of the RNA classifier and DNA‐RNA classifier between AUS/FLUS nodules and FN/SFN nodules (provided in Table S2). AUCs and confidence intervals (CIs) were assessed with medcalc version 18.2.1 (MedCalc Software, Ostend, Belgium). Expected PPV and NPV curves were based on calculated sensitivities and specificities over the range of the possible prevalence of malignancy (cancer or borderline lesion). All statistical analyses were performed with spss 17.0 (SPSS inc. Chicago, Illinois, USA), and *P* < 0.05 was considered statistically significant.

## 3 Results

### 3.1 Baseline characteristics of patients and nodules

We performed FNA for 2599 thyroid nodules from 2392 patients. Cytologically, 158 (6.1%) were reported as unsatisfactory samples, 377 (14.5%) were diagnosed as benign, 263 (10.1%) were classified as indeterminate, 354 (13.6%) were suspicious for malignancy and 1447 (55.7%) were malignant.

**Fig. 2:**
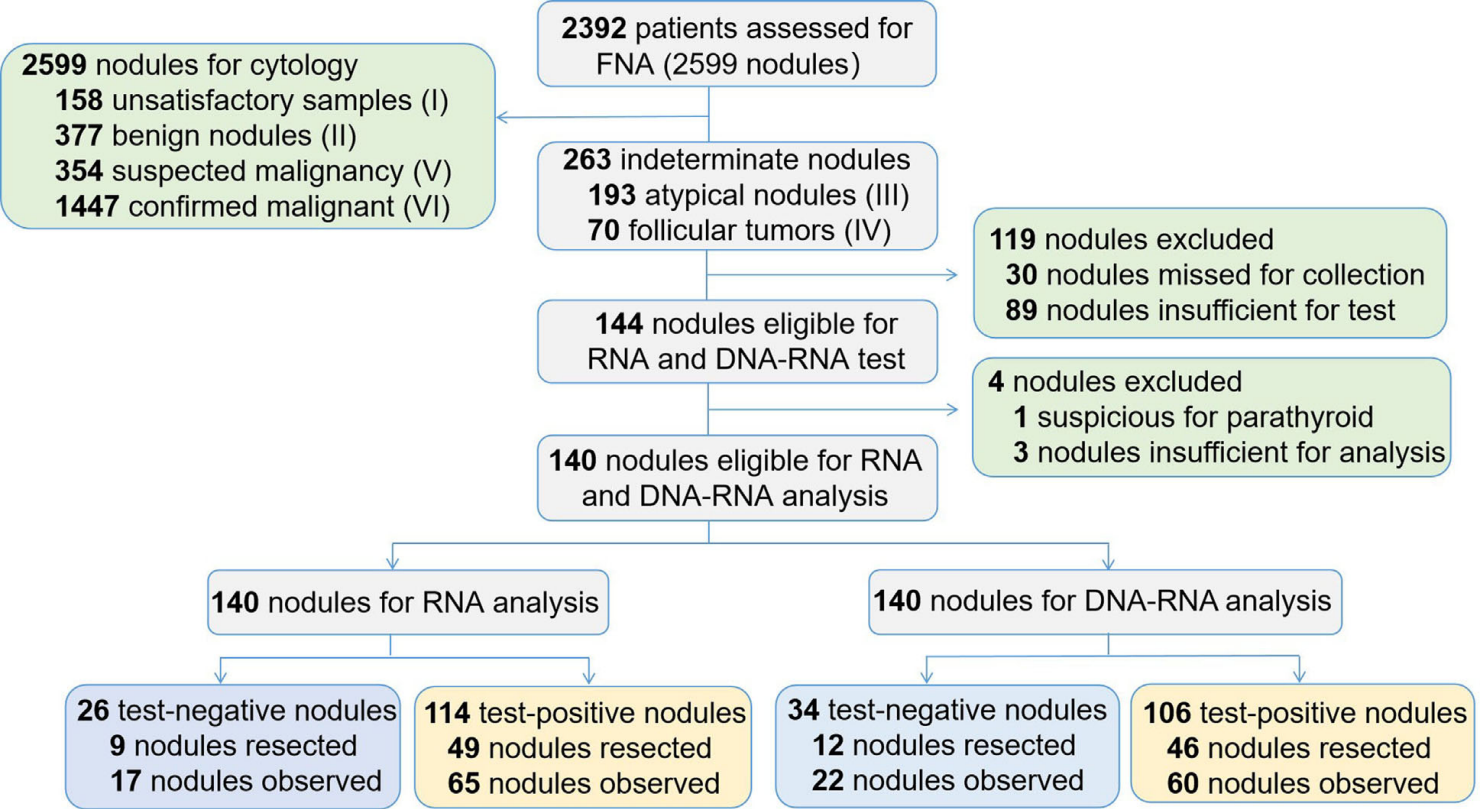
Study design and case selection. Nodules excluded: 119 indeterminate nodules were missed for collection, as 30 of them were missed out for collection of residual ThinPrep material by the cytotechnologist, and 89 residual liquids after routine ThinPrep were less than 10 mL and insufficient for subsequent test. Among cases subjected to molecular detection, four cases were not processed molecular tests, one suspicious for parathyroid and three insufficient for DNA or RNA. FNA, fine‐needle aspiration.

Of the 263 thyroid nodules with indeterminate results, 193 (73.4%) were classified as Bethesda III and 70 (26.6%) as Bethesda IV. A total of 119 indeterminate nodules were not included in subsequent molecular analysis due to insufficient samples (33.8%, 89/263) or missed collection by the cytotechnologist (11.4%, 30/263). Among 144 cases subjected to molecular detection, four cases were not processed molecular tests, one suspicious for parathyroid and three (2.1%, 3/144) insufficient for DNA or RNA. Ultimately, 140 samples from 136 patients were included in the ensuing statistical analysis (Fig. 2).

The characteristics of the patients and nodules are listed in Table 1. A total of 105 females (77.2%) and 31 males (22.8%) were included, with a median age of 48.5 (37.0–56.8) years. The median nodule size of 140 thyroid nodules was 1.0 (IQR, 0.7–1.5) cm.

**Table 1:** Baseline information for patients and nodules. IQR, interquartile range; AUS/FLUS, atypia of undetermined significance/follicular lesion of undetermined significance; FN/SFN, follicular neoplasm/suspicious for a follicular neoplasm.

Characteristic	*N* (%)
Patients, *N*	136
Age, median (IQR), years	48.5 (37.0–56.8)
Sex	
Female	105 (77.2)
Male	31 (22.8)
Nodules, *N*	140
Nodule size, cm	
Median (IQR)	1.0 (0.7–1.5)
0–1	72 (51.4)
>1–2	46 (32.9)
>2–4	16 (11.4)
>4	6 (4.3)
Bethesda System for Reporting Thyroid Cytopathology category	
III (AUS/FLUS)	87 (62.1)
IV (FN/SFN)	53 (37.9)
Hürthle cell predominance	13 (9.3)

### 3.2 No difference in resection rate between groups with positive or negative molecular testing results

RNA and DNA‐RNA molecular testing were simultaneously performed for all 140 samples with sufficient amounts of DNA and RNA. In the RNA molecular testing cohort, 26 (18.6%) indeterminate nodules were categorized as negative and 114 (81.4%) as positive. For nodules with negative RNA test results, nine (34.6%) were surgically resected; for nodules with positive RNA test results, 49 (43.0%) were resected. In the DNA‐RNA molecular testing cohort, 34 (24.3%) indeterminate nodules were categorized as negative and 106 (75.7%) as positive. For nodules with negative and positive DNA‐RNA test results, 35.3% (12/34) and 43.4% (46/106) were resected respectively. In both the RNA and DNA‐RNA test cohorts, the resection rates in the molecular positive group were slightly higher than those in the molecular negative group, but with no significant difference (p1 = 0.511 and p2 = 0.431). According to a retrospective study, the clinical management of cases with indeterminate cytology in our institute was based on ultrasound characteristics, clinical features and patient willingness rather than on molecular testing results. Our molecular results suggest that there is a considerable number of patients with indeterminate cytology and malignancy who do not choose surgery.

### 3.3 A high ROM value for resected indeterminate thyroid nodules

Among 140 indeterminate thyroid nodules, 58 (41.4%) were managed with surgical resection and subsequent histopathological analysis, of which 15 were benign (12 nodular goitre with adenomatous hyperplasia, two with Hashimoto's thyroiditis and one with adenoma), 10 borderline (three NIFTP and seven WT‐UMP) and 33 malignant (29 papillary thyroid carcinoma (PTC), one follicular carcinoma, two Hürthle cell carcinoma and one anaplastic carcinoma, Table 2). Hence, the ROM was 74.1% for resected indeterminate nodules, higher than that in North America, where a low ROM of 10–40% has been reported [10]. This highlights the difference in clinical practice for managing indeterminate nodules between our institution and North America.

**Table 2:** Histopathologic diagnosis of indeterminate thyroid nodules. NIFTP, non‐invasive follicular thyroid neoplasm with papillary‐like nuclear features; WT‐UMP, well‐differentiated tumour of uncertain malignancy.

Variable	Histopathologic diagnosis	Nodules with positive RNA test result	Nodules with positive DNA‐RNA test results	
	Total, *N* (%)	Total, *N* (%)	Total, *N* (%)	Gene alterations
Benign				
Hyperplasia	12 (20.7)	7 (14.3)	6 (13.0)	1 Nodule with NRAS; 1 HRAS
Thyroiditis	2 (3.5)	2 (4.1)	2 (4.3)	NA
Adenoma	1 (1.7)	0	0	NA
NIFTP	3 (5.2)	3 (6.2)	3 (6.5)	1 Nodule with BRAF K601E; 2 NRAS
WT‐UMP	7 (12.1)	5 (10.2)	3 (6.5)	1 Nodule with BRAF K601E; 1 NRAS
Papillary thyroid cancer				NA
Classic	9 (15.5)	9 (18.4)	9 (19.6)	3 Nodules with BRAF V600E; 1 KRAS; 1 NRAS; 2 ETV6/NTRK3 fusion
Classic + Follicular	3 (5.2)	3 (6.2)	2 (4.3)	1 Nodule with ETV6/NTRK3 fusion
Follicular	15 (25.9)	15 (30.6)	15 (32.6)	1 Nodule with BRAF V600E; 2 NRAS; 1 CCDC6/RET fusion; 1 ETV6/NTRK3 fusion; 1 NCOA4/RET fusion; 1 STRN/ALK fusion
Solid	1 (1.7)	1 (2.0)	1 (2.2)	NA
Oncocytic	1 (1.7)	1 (2.0)	1 (2.2)	1 Nodule with KRAS
Follicular carcinoma	1 (1.7)	1 (2.0)	1 (2.2)	NA
Hürthle cell carcinoma	2 (3.4)	1 (2.0)	2 (4.3)	1 Nodule with TERT
Anaplastic thyroid carcinoma	1 (1.7)	1 (2.0)	1 (2.2)	1 Nodule with TERT and BRAF V600E

### 3.4 RNA and DNA‐RNA classifiers show acceptable diagnostic ability in reclassifying indeterminate nodules

**Fig. 3:**
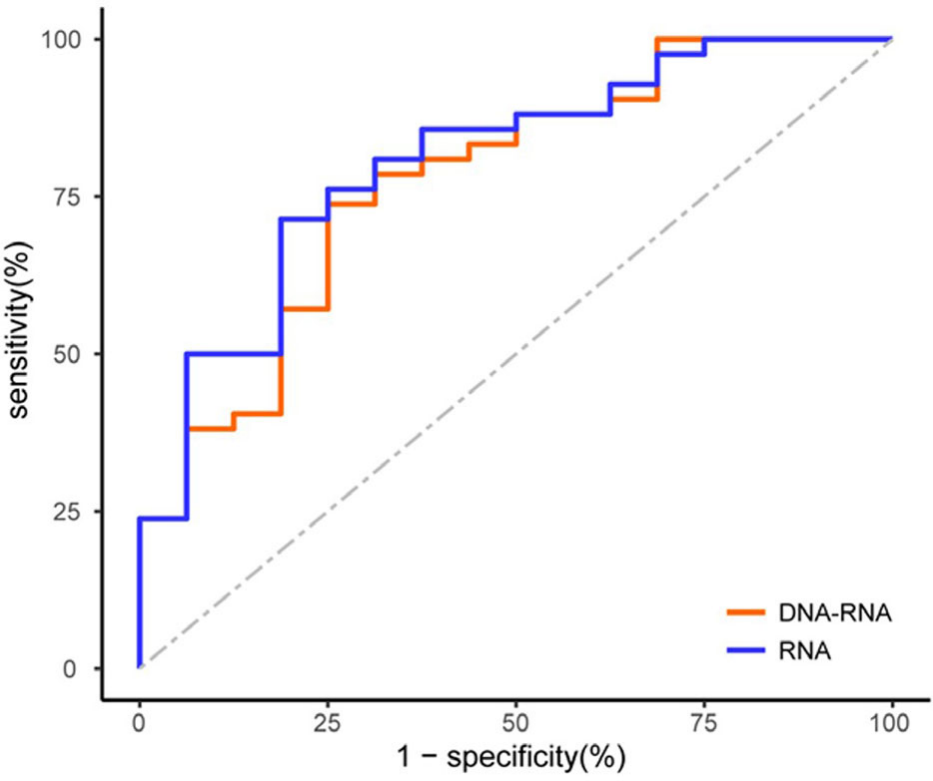
Receiver operating characteristic (ROC) curve for Thyroeva in malignant prediction of 58 resected indeterminate thyroid nodules.

The aim of this study was to distinguish histopathological malignant nodules from cytologically indeterminate nodules. To evaluate the diagnostic value of RNA and DNA‐RNA classifiers for predicting malignant nodules, we generated a receiver operating characteristic (ROC) curve. The AUC was 0.81 for the RNA classifier and 0.77 for the DNA‐RNA classifier, without a significant difference between them (*P* = 0.264) (Fig. 3). Hence, both classifiers showed acceptable diagnostic ability for reclassifying indeterminate nodules [24].

More specifically, the sensitivities were 93.0% (95% CI: 85.1–100.0%) for the RNA classifier and 88.4% (95% CI: 78.4–98.4%) (*P* = 0.458) for the DNA‐RNA classifier. The specificities were 40.0% (95% CI: 11.9–68.1%) for the RNA classifier and 53.3% (95% CI: 24.7–81.9%) (*P* = 0.464) for the DNA‐RNA classifier. For the RNA and DNA‐RNA classifier, the PPV was 81.6% (95% CI: 70.4–92.9%) and 84.4% (95% CI: 73.4–95.5%) (*P* = 0.717), and the NPV was 66.7% (95% CI: 28.2–100.0%) and 61.5% (95% CI: 30.9–92.1%) (*P* = 0.806) respectively (Table 3). Although previous trials have demonstrated that the DNA‐RNA test is more specific than the RNA test, a statistically significant difference in performance was not observed for our molecular test.

**Table 3:** Comparisons of published experiences and the present study. NIFTP, non‐invasive follicular thyroid neoplasm with papillary‐like nuclear features; AUS/FLUS, atypia of undetermined significance/follicular lesion of undetermined significance; FN/SFN, follicular neoplasm/suspicious for a follicular neoplasm; FNA, fine‐needle aspiration.

Author	Panel	No. of surgeries	Diagnostic performance (NIFTP = malignant)	*P*‐value
Present study	RNA	58	SN = 93.0% (40/43)	
			SP = 40.0% (6/15)	
			PPV = 81.6% (40/49)	
			NPV = 66.7% (6/9)	
Present study	DNA‐RNA	58	SN = 88.4% (38/43)	0.458^a^
			SP = 53.3% (8/15)	0.464
			PPV = 84.4% (38/45)	0.717
			NPV = 61.5% (8/13)	0.806
Alexander EK (2012)	GEC	210	SN = 90.2% (46/51)	0.625^b^
			SP = 51.6% (82/159)	0.391
			PPV = 37.4% (46/123)	0.000
			NPV = 94.3% (82/87)	0.004
Livhits MJ (2020)	ThyroSeq v3	60	SN = 96.9% (31/32)	0.179^c^
			SP = 35.7% (10/28)	0.264
			PPV = 63.3% (31/49)	0.02
			NPV = 90.9% (10/11)	0.098

a RNA results of the present study vs. DAN‐RNA results of the present study.

b RNA results of the present study vs. GEC results of Alexander EK.

c DNA‐RNA results of the present study vs. ThyroSeq v3 results of Masha. Performance comparisons were carried out using the chi‐squared test.

In the RNA molecular test cohort, nine false positive and three false negative test results occurred, the latter included two patients with WT‐UMP and one with Hürthle cell carcinoma. In the DNA‐RNA cohort, eight were false positive and five were false negative tests, including four cases of WT‐UMP and one case of PTC. Altogether, false negative cases based on RNA or DNA‐RNA testing largely involved borderline lesions diagnosed by histological examination. The deficiency of molecular testing for borderline lesions was mainly due to their highly heterogeneous transcriptomic landscape, which increases the difficulty of gene expression‐based classification [25]; false negative test results also occurred due to a high level of disagreement between pathologists regarding the diagnosis of borderline lesions. In fact, some false negative results may not be authentically false but caused by disagreement in histological diagnosis.

### 3.5 RNA and DNA‐RNA classifiers demonstrate higher PPV and may severe as a ‘rule‐in’ test for indeterminate thyroid nodules

Compared with Afirma GEC, the RNA classifier had a higher PPV (*P* = 0.000) and a lower NPV (*P* = 0.004), but no significant differences was observed for sensitivity (*P* = 0.625) or specificity (*P* = 0.391). Similarly, the DNA‐RNA classifier showed a higher PPV (*P* = 0.020) and a lower NPV (*P* = 0.098) than ThyroSeq v3, with no significant difference in sensitivity (*P* = 0.179) or specificity (*P* = 0.264) (Table 3). Based on the higher PPV and lower NPV, both the RNA and DNA‐RNA classifiers may serve as a ‘rule‐in’ test for risk stratification of indeterminate nodules.

**Fig. 4:**
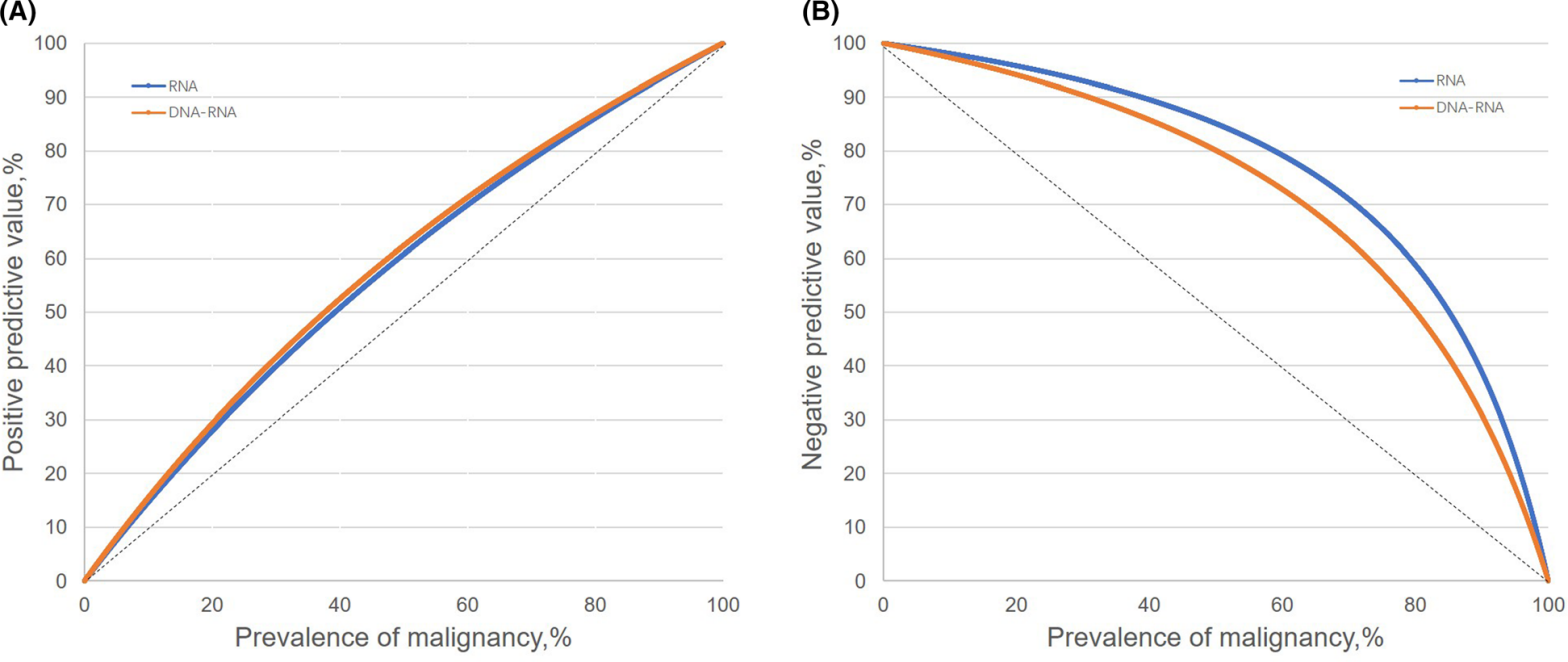
Expected positive and negative predictive value curves for Thyroeva in 58 resected indeterminate thyroid nodules. (A) Expected positive predictive value curves of molecular testing. The blue line indicates the RNA classifier, and the orange line indicates the DNA‐RNA classifier. (B) Expected negative predictive value curves of molecular testing. The blue line indicates the RNA classifier, and the orange line indicates the DNA‐RNA classifier.

Based on the sensitivities and specificities calculated over the range of possible prevalence of malignancy (cancer or borderline lesion) for indeterminate thyroid nodules, we generated an expected PPV curve as well as an expected NPV curve for our molecular tests (Fig. 4). In Fig. 4, the PPV of RNA classifier was similar to that of DNA‐RNA classifier, whereas the NPV of RNA classifier is higher than that of DNA‐RNA classifier. The predictive value of the RNA or DNA‐RNA classifier was affected by the prevalence of malignancy in indeterminate nodules. Based on the high ROM value of 74.1%, molecular testing is suggested as a ‘rule‐in’ test for our institution. When the ROM decreased to 30.9% or less, both the RNA and DNA‐RNA classifiers showed a high NPV (≥ 90%); thus, the ‘rule‐out’ test may be more appropriate. Overall, the application mode of ‘rule‐in’ or ‘rule‐out’ for molecular testing should depend on the ROM of different regions and populations.

### 3.6 DNA‐RNA testing provides more specific molecular alterations than RNA testing

**Fig. 5:**
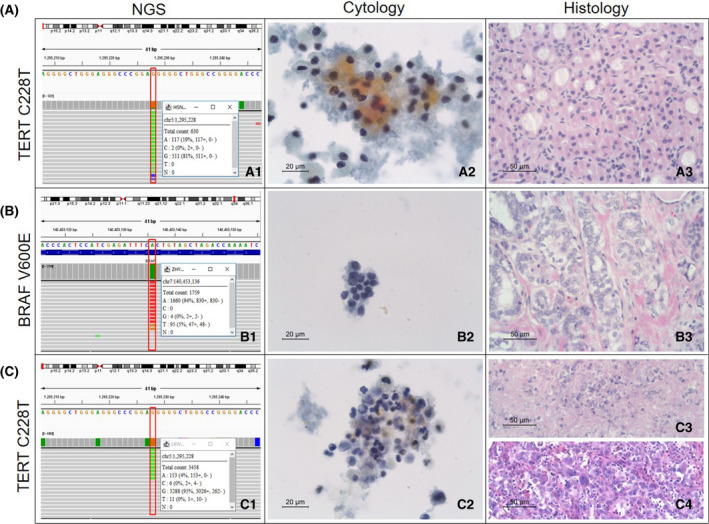
Correlation of cytological and histological morphology and molecular results in 58 resected indeterminate thyroid nodules. (A) Hürthle cell carcinoma with cytological diagnosis of Bethesda IV. (A1) Molecular testing showing TERT mutation. (A2) Cytopathology, this cellular specimen consisted of dispersed Hürthle cells with highly variable size and abundant and finely granular cytoplasm. (ThinPrep^®^, Papanicolaou stain, ×400). (A3). Histopathology, thyroidectomy section (HE stain, ×200). (B) A classic and follicular variant of thyroid papillary carcinoma with cytological diagnosis of Bethesda III. (B1) Molecular testing showing BRAF mutation (B2) Cytopathology, a low‐cellular specimen composed of cells with slight atypia, showing nuclear enlargement and slight chromatin pallor. The atypical cells were insufficient for a diagnosis of suspicious malignancy. (ThinPrep^®^, Papanicolaou stain ×400) (B3). Histopathology, thyroidectomy section, area of follicular variant (HE stain, ×200). (C) Anaplastic thyroid carcinoma with cytological diagnosis of Bethesda IV. (C1) Molecular testing showing TERT mutation (BRAF and TERT mutations were detected simultaneously in this anaplastic carcinoma; the BRAF mutation is not shown). (C2) Cytopathology: a specimen composed of a few epithelial cells. Some of the nuclei were deeply stained, demonstrating degenerative changes. Neutrophils and unstructured blue substances are shown in the background (ThinPrep^®^, Papanicolaou stain ×400). (C3). Histopathology, thyroidectomy section, area of necrosis (HE stain, ×200). (C4). Histopathology, thyroidectomy section, tumour (HE stain, ×200).

In the DNA‐RNA testing cohort, 26 DNA mutations and fusions were detected by molecular analysis, as shown in Table 2, including five cases of BRAF V600E mutation, two of BRAF K601E mutation, 10 of RAS mutation, two of TERT mutation and seven gene fusions (one CCDC6‐RET, one NCOA4‐RET, four ETV6_NTRK3 and one STRN‐ALK). All five nodules harbouring the BRAF V600E mutation were malignant carcinomas, including three classic papillary thyroid carcinomas, one follicular variant papillary carcinoma and one anaplastic thyroid carcinoma concurrent with TERT mutation (Fig. 5). Two nodules with TERT mutation detected in our study were also associated with malignancy, which included anaplastic thyroid carcinoma and Hürthle cell carcinoma. Additionally, the seven gene fusions detected in indeterminate nodules are all associated with malignancy.

Nevertheless, RAS or RAS‐like mutations (e.g. BRAF K601E) are not specifically associated with malignancy and are usually detected in follicular‐patterned neoplasms, which commonly include follicular adenoma, NIFTP, follicular variant PTC and follicular carcinoma. Among our 10 nodules with RAS mutations, two were benign hyperplasia (one NRAS and one HRAS), two were NIFTP (two NRAS), one was WT‐UMP (NRAS) and five were PTC (three NRAS and two KRAS). When RAS mutations were identified alone, the probability of cancer, NIFTP or WT‐UMP was 80%. Two cases of BRAF K601E mutation were detected: one in a NIFTP nodule and the other in a WT‐UMP.

The expanded gene mutation panel of the DNA‐RNA test contributed greatly to the diagnosis of thyroid cancer based on the specific molecular alteration detected (e.g. BRAF V600E), which is important for a ‘rule‐in’ test.

## 4 Discussion

Fine‐needle aspiration is now the most successful diagnostic test in the management of thyroid nodules. Nonetheless, approximately 10–40% of FNAs are not conclusively diagnosed by cytology and are categorized as indeterminate [4, 26, 27, 28]. ATA guidelines suggest that molecular testing may be used to evaluate malignancy risk, and many practices have incorporated molecular testing as part of the work‐up for an indeterminant nodule [11, 12, 13]. However, molecular testing has seldom been studied in regions outside North America, including Asia. It is also important to highlight that no specific molecular product or test is considered to be the gold standard, and thus, it is challenging to make a generalization.

In the current study, a ROM of 74.1% was calculated for indeterminate nodules, which is much higher than that reported for indeterminate categories in North America, at 10–40% [11, 12]. One explanation for the high rate of ROM is that Asians and clinicians tend to consider false positive results more significantly than false negative results. Furthermore, cytopathologists may have a tendency to classify cases with equivocal PTC nuclear features into the indeterminate category (Bethesda III), whereas many of these nodules are diagnosed as ‘suspicious’ by Western cytopathologists [29, 30]. Another factor that may impact ROM is the prevalence of disease in a given patient cohort. In China, FNA is recommended only for nodules with high‐ and intermediate‐suspicion sonographic patterns [31], but not for low‐suspicion nodules (size ≥ 1.5 cm) and very low‐suspicion nodules (size ≥ 2.0 cm) as recommended in ATA guidelines [5]. In general, the criteria for FNA leads to a high proportion of malignant FNA nodules. As in the present study, 55.7% of FNA nodules were categorized as malignant and 13.6% as suspicious of malignancy, and both categories were much higher than those recommended in the Bethesda guidelines from North America (1–6% and 2–5% respectively) [2]. Despite strict triage of patients with indeterminate nodules in Asian countries has been suggested to be responsible for a low resection rate and a high ROM of surgically treated indeterminate nodules [10, 20], the effect of strict triage for surgery based on the value of ROM in surgically treated indeterminate nodules did not seem to be significant in our data. The resection rates of indeterminate nodules in the molecular test‐positive group were slightly higher than those in the molecular test‐negative group, but no significant difference was found. Therefore, a considerable number of patients with indeterminate cytology and malignancy might not choose surgery. In other words, the selection of surgery based on ultrasound characteristics, clinical features and patient willingness in our clinical practice is not very effective, and more information is needed for decision‐making, such as the results of molecular testing.

Given that no commercial molecular test for indeterminate thyroid nodules approved by the FDA is available in China, we developed a novel molecular platform that included both RNA and DNA‐RNA sequencing tests, corresponding to the commercial Afirma GEC and ThyroSeq v3 tests respectively. With regard to reclassifying indeterminate thyroid nodules, the AUCs for the RNA and DNA‐RNA classifiers were 0.81 and 0.77, respectively, showing an acceptable ability to identify thyroid carcinoma among indeterminate thyroid nodules. To better evaluate the diagnostic ability of the novel platform, we performed statistical analysis between our results and those reported in North America [11, 13]. As shown in Table 3, no significant difference in specificity or sensitivity was observed for either the RNA or DNA‐RNA classifier. Therefore, our novel molecular platform of both RNA and DNA‐RNA sequencing testing is as effective as commercial RNA (Afirma GEC) and DNA‐RNA (ThyroSeq v3) tests.

Nevertheless, in our cohort, the PPVs of the RNA and DNA‐RNA classifiers were higher (81.6% and 84.4% vs. 37.4% and 63.3%; p1 = 0.000 and p2 = 0.02) and the NPVs were lower (66.7% and 61.5% vs. 94.3% and 90.9%; p1 = 0.004 and p2 = 0.098) than those of Afirma GEC and ThyroSeq v3 respectively [11, 13]. The discrepancy in the predictive values might be due to differences in molecular test performance. As a high PPV and low NPV were found, the RNA and DNA‐RNA classifiers may be suggested to be ‘rule‐in’ tests. More importantly, our institute performs strict management for indeterminate nodules, with decision for surgery based on ultrasound characteristics, clinical features and patient willingness, and a ‘rule‐in’ test is more useful for such decision‐making.

According to the expected PPV and NPV curves for our novel molecular platform, the application mode of ‘rule‐in’ for RNA and DNA‐RNA testing is determined by the high ROM of the indeterminate nodules we examined. When the ROM value decreased to 30.9% or less, both classifiers may be suggested as ‘rule‐out’ tests. If molecular testing is performed as ‘rule‐out’ test, RNA classifier is superior to DNA‐RNA classifier for the higher NPV and cheaper cost. In general, the application mode of ‘rule‐in’ or ‘rule‐out’ for molecular testing depends on the ROM in different regions and populations.

Although no statistically significant differences in diagnostic capacity between the RNA and DNA‐RNA classifiers was observed, the expanded gene mutation revealed by DNA‐RNA testing may be valuable for diagnosing thyroid cancer based on specific molecular alterations. Particularly when molecular testing is used as a ‘rule‐in’ test, characteristic high‐risk mutations will directly lead to a decision of surgery for indeterminate nodules. Both our current and previous studies show that TERT and BRAF V600E mutations and gene fusions are associated with a very high probability of malignancy [32, 33, 34, 35, 36]. In the present cohort, 39.4% of thyroid cancers with these high‐risk mutations were detected, including five cases of BRAF V600E mutation (one combined with TERT mutation), two cases of TERT mutations and seven gene fusions. These high‐risk genetic changes may play a key role in surgery decision‐making.

RAS or RAS‐like mutation (e.g. BRAF K601E) is not specifically associated with malignant outcomes, with an approximately 40–80% probability of cancer or NIFTP [16, 32, 35]. Therefore, it is insufficient for surgeons to make a decision regarding surgery for indeterminate nodules when only RAS or RAS‐like mutation is identified. Indeed, the therapeutic strategy will depend on the patients’ willingness and ultrasound results, as they are equally important. We detected 10 cases of RAS mutation and two of BRAF K601E mutation in the present cohort, and the probability of cancer, NIFTP or WT‐UMP for RAS mutation is 80%. The two cases of BRAF K601E mutation were both in borderline tumours: one for NIFTP and the other for WT‐UMP. This was in line with a previous report that the BRAF K601E mutation is strongly associated with follicular‐patterned cancer, particularly with the encapsulated follicular variant of PTC [37, 38, 39]. Thus, when molecular testing is performed as ‘rule‐in’ test in clinical practice, expanded DNA‐RNA testing may be more valuable than RNA testing alone.

One advantage of the present study is that we used residual ThinPrep^®^ material to perform molecular testing, which was different from a previous experimental design [40] in which patients undergoing molecular testing underwent puncture twice: one for cytology evaluation and another for molecular testing. In fact, using residual ThinPrep^®^ material, a single puncture is sufficient for both cytological diagnosis and molecular testing because the liquid‐based sample remaining requires no additional FNA, reducing the burden on patients and saving medical resources. In the current study, among the indeterminate FNA results, residual liquid‐based samples for further molecular testing were available for 54.8% of patients, 97.8% of them having sufficient RNA and DNA to carry out molecular testing. Therefore, we strongly recommend using residual liquid‐based samples for molecular testing when available.

There are some limitations in our study. First, as no commercial Afirma GEC and ThyroSeq v3 tests are available in China, we could not directly compare the performance of these two tests between regions. Second, there were some histopathological borderline tumours in our study that were diagnosed with a high level of disagreement between pathologists [41, 42]. Our false negative cases based on RNA or DNA‐RNA detection largely involved borderline lesions: of the three false negative cases by our RNA test, two were borderline tumours; and of the five false negative cases by our DNA‐RNA test, four were borderline tumours. Overall, the diagnostic capacity of molecular testing may be underestimated due to the uncertainty of the diagnostic gold standard (an analysis considering borderline lesions as benign is provided as Supplement). Third, considering molecular testing was proceeded by using residual ThinPrep^®^ FNA samples, more nodules categorized as AUS/FLUS were excluded as there were fewer cells in the AUS/FLUS residues compared to those categorized as FN/SFN. Hence, the proportion of FN/SFN nodules among indeterminate nodules based on molecular testing was higher than that among all indeterminate nodules. Finally, because our current study is a retrospective analysis, the clinical management of patients was not based on molecular testing results. A large number of cases with positive molecular test results did not undergo surgery; as such, next‐step follow‐up and/or prospective studies should be carried out. Moreover, the application of molecular testing in our clinical practice should be strengthened.

## 5 Conclusion

In regions with high ROM of indeterminate nodules, RNA and DNA‐RNA tests may serve as ‘rule‐in’ tests in our clinical practice, especially an expanded DNA‐RNA testing may be more valuable than RNA testing alone. Whereas, in regions with low ROM, RNA and DNA‐RNA tests may serve as ‘rule‐out’ tests and a single RNA test is enough. In one word, the performance mode of ‘rule‐in’ or ‘rule‐out’ for molecular testing correlates closely with the ROM value of indeterminate thyroid nodules. In addition to the difference in thyroid cancer practice between Western countries and Asia highlighted in our study, it should be noted that there is still a considerable amount of variability among countries on the same continent as well as among national institutions. It remains challenging to define a universally acceptable approach of molecular testing for indeterminate thyroid nodules.

## Conflict of interest

The authors declare no conflict of interest.

## Author contributions

CH and WJ contributed equally to this work. CH reviewed the histopathological sections of the resected thyroid samples and wrote the manuscript. WJ carried out the data acquisition and assisted in writing the original draft. QC performed the ultrasonic diagnosis. ZZ and JC performed the cytological diagnosis. LZ prepared the cytological sections. CW collected the cytological samples. YS and ZL carried out the RNA and DNA extractions. HZ performed the cytological diagnosis and data analysis. TX conducted the molecular detection and data analysis. GH obtained the financial support and designed and conducted this study. All authors approved the final version of the manuscript.

### Peer review

The peer review history for this article is available at https://publons.com/publon/10.1002/1878‐0261.13205.

## Supporting information


**Table S1.** Diagnostic performance of molecular tests considering borderline lesions as benign. The ROM of indeterminate nodules and the diagnostic performance of molecular tests were analysed considering borderline lesions as benign. An additional comparations of the diagnostic performance of the RNA classifier and DNA‐RNA classifier between Bethesda III and Bethesda IV nodules were performed.
**Table S2.** Diagnostic performance of molecular tests considering borderline lesions as malignant. The ROM of indeterminate nodules and the diagnostic performance of molecular tests were analysed considering borderline lesions as malignant. An additional comparations of the diagnostic performance of the RNA classifier and DNA‐RNA classifier between Bethesda III and Bethesda IV nodules were performed.

## Data Availability

The data are not available in a public database or repository. The datasets generated and analysed in this study are available from the corresponding author on reasonable request.
