# Supplementary material for: Risk stratification of indeterminate thyroid nodules by novel multigene testing: a study of Asians with a high risk of malignancy
Source: Mol Oncol. 2022 Mar 12;16(8):1680–93. doi: 10.1002/1878-0261.13205 (PMC9019878; doi:10.1002/1878-0261.13205)
Supplement: Supplementary file 1 — Table S1. Diagnostic performance of molecular tests considering borderline lesions as benign. The ROM of indeterminate nodules and the diagnostic performance of molecular tests were analysed considering borderline lesions as benign. An additional comparations of the diagnostic performance of the RNA classifier and DNA‐RNA classifier between Bethesda III and Bethesda IV nodules were performed. Table S2. Diagnostic performance of molecular tests considering borderline lesions as malignant. The ROM of indeterminate nodules and the diagnostic performance of molecular tests were analysed considering borderline lesions as malignant. An additional comparations of the diagnostic performance of the RNA classifier and DNA‐RNA classifier between Bethesda III and Bethesda IV nodules were performed. [file MOL2-16-1680-s001.docx]

Supplemental table 1. Diagnostic performance of molecular tests considering borderline lesions as benign.

| Bethesda  category | ROM | Panel | | | | | | | | |
| --- | --- | --- | --- | --- | --- | --- | --- | --- | --- | --- |
|  |  | RNA | | | |  | DNA-RNA | | | |
|  |  | SN | SP | PPV | NPV |  | SN | SP | PPV | NPV |
| III | 57.6%(19/33) | 100.0(19/19) | 35.7(5/14) | 67.9(19/28) | 100.0(5/5) |  | 100.0(19/19) | 42.9(6/14) | 70.4(19/27) | 100.0(6/6) |
| IV | 60.0%(15/25) | 93.3(14/15) | 30.0(3/10) | 66.7(14/21) | 75.0(3/4) |  | 93.3(14/15) | 60.0(6/10) | 77.8(14/18) | 85.7(6/7) |
| p value |  | 0.253 | 0.77 | 0.93 | 0.236 |  | 0.253 | 0.408 | 0.582 | 0.335 |
| III+IV | 58.6%(34/58) | 97.1(33/34) | 33.3(8/24) | 67.3(33/49) | 88.9(8/9) |  | 97.1(33/34) | 50.0(12/24) | 73.3(33/45) | 92.3(12/13) |
| p value* |  |  |  |  |  |  | 1 | 0.242 | 0.526 | 0.784 |

*RNA results vs. DNA-RNA results in categories III and IV.

Supplemental table 2. Diagnostic performance of molecular tests considering borderline lesions as malignant.

| Bethesda  category | ROM | Panel | | | | | | | | |
| --- | --- | --- | --- | --- | --- | --- | --- | --- | --- | --- |
|  |  | RNA | | | |  | DNA-RNA | | | |
|  |  | SN | SP | PPV | NPV |  | SN | SP | PPV | NPV |
| III | 66.7%(22/33) | 95.5(21/22) | 36.4(4/11) | 75.0(21/28) | 80.0(4/5） |  | 95.5(21/22) | 45.5(5/11) | 77.8(21/27) | 83.3(5/6) |
| IV | 84.0%(21/25) | 90.5(19/21) | 50.0(2/4) | 90.5(19/21) | 50.0(2/4) |  | 81.0(17/21) | 75.0(3/4) | 94.4(17/18) | 42.9(3/7) |
| p value |  | 0.522 | 0.634 | 0.166 | 0.343 |  | 0.138 | 0.31 | 0.131 | 0.135 |
| III+IV | 74.1%(43/58) | 93.0% (40/43) | 40.0% (6/15) | 81.6% (40/49) | 66.7% (6/9) |  | 88.4% (38/43) | 53.3% (8/15) | 84.4% (38/45) | 61.5% (8/13) |
| p value* |  |  |  |  |  |  | 0.458 | 0.464 | 0.717 | 0.806 |

*RNA results vs. DNA-RNA results in categories III and IV.
